# Studies on the deposition of copper in lithium-ion batteries during the deep discharge process

**DOI:** 10.1038/s41598-021-85575-x

**Published:** 2021-03-18

**Authors:** Thomas Langner, Tim Sieber, Jörg Acker

**Affiliations:** Department of Physical Chemistry, Brandenburg University of Technology Cottbus-Senftenberg, 01968 Senftenberg, Germany

**Keywords:** Chemistry, Energy science and technology, Materials science

## Abstract

End-of-life lithium-ion batteries represent an important secondary raw material source for nickel, cobalt, manganese and lithium compounds in order to obtain starting materials for the production of new cathode material. Each process step in recycling must be performed in such a way contamination products on the cathode material are avoided or reduced. This paper is dedicated to the first step of each recycling process, the deep discharge of lithium-ion batteries, as a prerequisite for the safe opening and disassembling. If pouch cells with different states of charge are connected in series and deep-discharged together, copper deposition occurs preferably in the cell with the lower charge capacity. The current forced through the cell with a low charge capacity leads, after lithium depletion in the anode and the collapse of the solid-electrolyte-interphase (SEI) to a polarity reversal in which the copper collector of the anode is dissolved and copper is deposited on the cathode surface. Based on measurements of the temperature, voltage drop and copper concentration in the electrolyte at the cell with the originally lower charge capacity, the point of dissolution and incipient deposition of copper could be identified and a model of the processes during deep discharge could be developed.

## Introduction

Lithium-ion batteries (LiBs) are currently the most important technology for storing electrical energy and increasingly penetrating all areas of human everyday life due to their expanding use in smartphones, laptops, tools and e-mobility. Commercial cells are usually manufactured in a cylindrical design, whereas LiBs are used in the automotive industry in large prismatic cells or as so-called pouch cells.

At the end of their lifetime, LiBs represent a valuable secondary raw material source to cover the demand of the necessary elements (Li, Ni, Mn, Co). Recycling is becoming increasingly important as many such batteries are at the end of their life cycle and the raw materials are expensive and are mined under critical conditions. There are currently two basic industrially implemented technologies for the recycling of spent LiBs: the pyrometallurgical and the hydrometallurgical process^1–11^. The pyrometallurgical route is energy-intensive due to the high temperatures required. After pretreatment, cobalt and manganese nickel are separated pyrometallurgically from the spent LiBs by melting and refining. The hydrometallurgical route involves leaching disassembled or shredded LiBs in strong inorganic acids to dissolve metals and battery materials. Similar to the pyrometallurgical method, the metals are recovered as inorganic salts. Losses typically occur during leaching and precipitation/separation of the metal salts. In addition, the complete production chain (precipitation, calcination, shaping, tempering) must then be run through again.

Sieber et al. chose an alternative approach for the recovery of nickel, manganese and cobalt (NMC), which is carried out while maintaining the chemical, physical and morphological properties of the NMC and with the minimal use of chemicals^12^. This approach, known as functional recycling, can be applied to the cathodes of disassembled and separated LiBs at the end of their lifetimes and to residues from cathode production. The target of functional recycling is the recovery of high quality cathode material which can be reused for the production of new LiBs, so-called recycled batteries. Functional recycling takes into account the current development that the performance and, thus, the added value of modern high-performance materials is determined significantly by their defined composition, the effort and know-how of their synthesis or their defined geometric shape, e.g. as fibers, hollow fibers, micro- and nanowires, defined layers and stratifications, and particles of a defined size. Therefore, all process steps of functional recycling must be designed in a way that changes in the chemical composition of the materials used or the topographies of the surfaces of the compounds are avoided or minimized.

The first step in recycling is always the deep discharge of the modules, in which the individual battery cells (e.g. pouch, prismatic) are electrically combined into a single unit, so that the lithium is transferred to the cathode material as completely as possible to ensure that the cells can be dismantled safely. This is followed by dismantling the batteries, opening the cells and separating the anode, cathode and separator foils. All further steps of the chemical–mechanical separation of the cathode material from the aluminum foil must also be designed in such a way that any degradation of the particles, for example, due to breakage, deposits on the surface, contamination with other battery components (e.g. particles of the aluminum or copper foils) or direct chemical attack on the material is prevented or minimized.

The subject of this study is the first step of recycling, the deep discharge of the battery, which has an enormous impact on the functional recycling and reusability of the cathode material recovered.

An unwanted side reaction that can occur during deep discharge is the deposition of copper on the cathode foil. Jo et al. found out in their investigations that an increasing copper content in the NMC leads to a loss of capacity of the battery^13^. They found a slightly lower discharge capacity at a copper content of 0.5…1.5 mol%. After 50 cycles, the capacity of the pure active material was 135.64 mAh g^−1^. By comparison, the discharge capacity of the active material contaminated with 0.5, 1.5 and 2.5 mol% was 131.81, 129.17 and 85.2 mAh g^−1^, respectively. The material contaminated with 2.5 mol% copper showed a slightly lower capacity than the pure active material only at low discharge rates (0.1 C). At high discharge rates (5 C), the capacity decreases by about 85% compared to cells with copper-free cathode material.

Guo et al. connected four fully charged NMC-based batteries (state of charge: SOC = 100%) in series with a fully discharged battery (SOC = 0%)^14^. The current flow generated when the full battery is discharged forces a charge transport on the discharged cell, which, due to the absence of Li^+^, must be ensured by other charge carriers as an unwanted side reaction. They found that the voltage curve of the initially deeply discharged cell passes through a minimum at a SOC = − 11% (relative to the initially fully charged batteries) during further “discharging.” The time the voltage reaches the minimum was declared as the point at which the dissolution of the copper foil of the anode begins. According to Guo, copper is deposited on the cathode at the interface to the separator and leads to local short circuits from a SOC = − 13% onward, which increase in frequency up to SOC = − 20%, and the internal resistance of the cell asymptotically approaches a limit value. The process can be reversed as long as the dissolved copper (SOC > − 12%) has not yet been deposited on the cathode. These cells can be almost fully recharged and show only slight power losses. Cells that were discharged to a SOC ≤ − 14.5% and deposited copper could be charged but showed a significant self-discharge and depleting open-circuit voltage.

Zheng et al. investigated the degradation mechanisms of LiFePO_4_ cells as a function of overdischarge^15^. They found a correlation between the capacity loss of the cell and the value of the end-of-discharge voltage. They found that overdischarge to 0.5 V and 0 V lead to lower cycle performance in addition to serious capacity loss. Electrode impedance trends showed that the impedance of both electrodes increased, with the cell at 0 V exhibiting the highest values. Based on half-cell tests, the capacity loss could be related to the anode.

Experiments on overdischarge were carried out by Fear et al. with NCA (nickel–cobalt-aluminum oxide) as the cathode material^16^. They divided the discharge into different phases using the first and second derivation of the voltage curve. They concluded that the oxidation of the copper of the carrier foil begins at the minimum voltage curve at − 1.5 V, where the first derivative is 0, and is followed by the dissolution of the copper after the NCA breakdown. Furthermore, they described the voltage rise after the minimum by the increasing potential of the cathode, since the overpotential for copper reduction is reduced and copper ions compete with the lithium ions to be reduced at the electrode surface, as already described by Kasnatscheew^17^. The internal resistance of the cell decreases due to internal short circuits and the voltage approaches − 0.23 V asymptotically. At this point, the copper bridges across the cell have grown sufficiently so that the cell behaves like a resistor in the circuit rather than an electrochemical system.

Robles et al. performed long-term cycling to investigate the degradation mechanisms as a function of overdischarge^18^. Cells whose discharge voltage was 2.7 V exhibited a 20% capacity loss after 287 cycles. The capacity loss was attributed to the thickening of the SEI. If the discharge voltage is set to of 1.5 V, the capacity loss of 20% is reached after 120 cycles. In addition to SEI thickening, increased li-plating is responsible for this. If the final discharge voltage is set to 0 V or − 0.5 V, Li-plating, particle cracking, copper dissolution and the formation of copper bridges occur. Cells that were overdischarged to this − 0.5 V failed after only 14 cycles due to internal short circuits.

The deep discharge of LiCoO_2_ cells to 0 V leads to an increase of the anode potential to about 3.5 V, so that the copper of the anode foil is oxidized and, as a result, is deposited on the cathode foil. The investigations of Li et al. showed that the SEI decomposes to gaseous products (CO, CO_2_, CH_4_) and the cells swell during deep discharge to 0 V^19^. Kasnatscheew et al. analyzed the interactions between the electrodes in a three-electrode Swagelok cell during overdischarge by determining the single-electrode potentials of anode and cathode to a reference electrode located in the system^17^. A characteristic potential plateau at about 3.56 V was found at the graphite electrode due to the oxidation of the copper. The constant anode potential after the beginning of the copper oxidation was interpreted to mean that this process continues during the entire remaining discharge phase. The time-delayed potential drop observed at the positive electrode was attributed to the competitive reaction between the conventional lithium plating reaction and the parasitic Cu plating reaction. He et al. cyclized different LiFePO_4_ cells under overdischarge conditions (5, 10, 15 and 20% overdischarge)^20^. Under these conditions, for example, the cell cyclized to 120% depth of discharge failed on the second cycle. The oxidation and reduction potentials of Cu/Cu^+^ and Cu^+^/Cu^2+^ were determined vs. Li/Li^+^. They showed that there is a gradual formation of copper bridges, which lead to internal short circuits and result in considerable self-discharge.

Hendricks et al. performed deep discharges to 0.5 V, 0.25 V and 0 V^21^. The electrodes were subsequently examined with XPS and XAFS. Copper was detected in all cells discharged below 0.5 V, which was attributed to the dissolution of the anode current collector. They suggested that the dissolution of the copper foil leads to a poorer adhesion of the anode material, which justifies a capacity loss, during 40 cycles, of 10%. Furthermore, they found that the deposition of copper species leads to a blockage of intercalation sites and thus also contributes to a capacity loss. The deposited copper species were identified as Cu_2_O and Cu(OH)_2_. These species are non-conductive and thus do not lead to internal short circuits. However, it was not been excluded that overdischarge into reversal could result in deposition of metallic copper.

It is necessary for the best possible performance of the recyclate that the material for recycling is copper-free; this applies to both the active material and the electrolyte. It is, therefore, crucial to determine the point on the voltage curve during discharge where copper is present in the electrolyte and the point where copper is deposited.

## Results

### Discharge of individual cells

**Figure 1:**
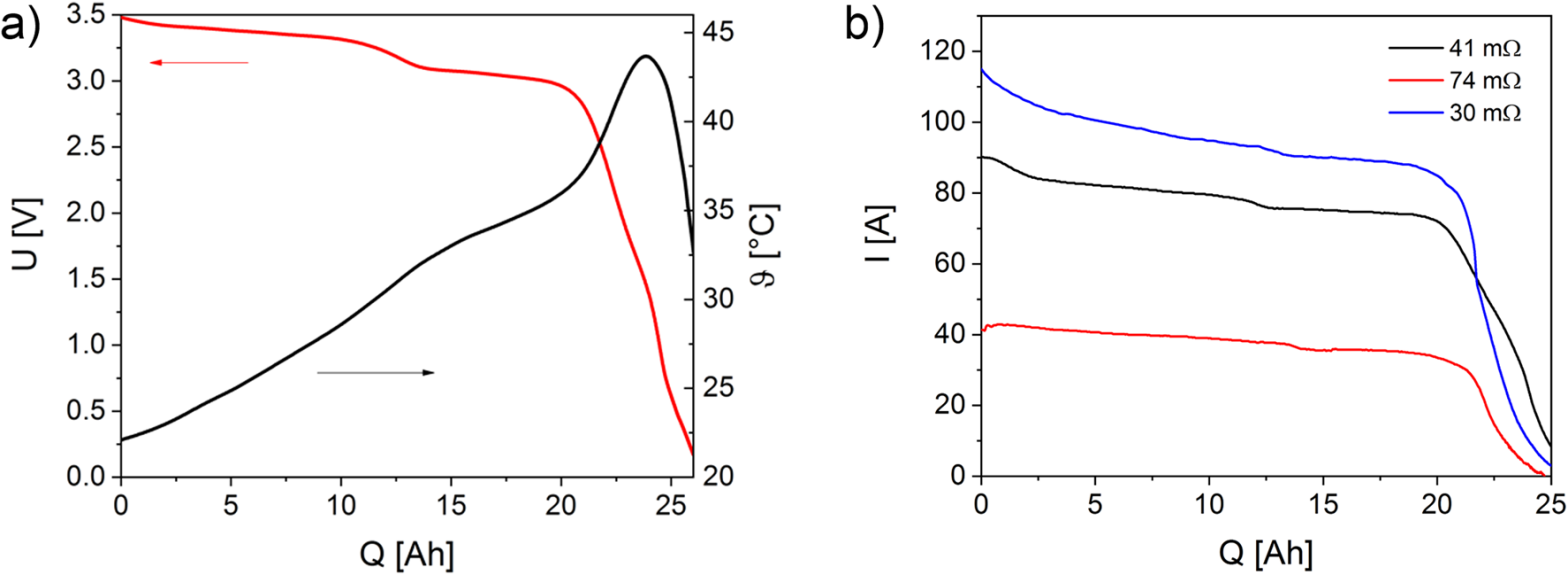
(**a**) Voltage and temperature behavior during a deep discharge of a single cell at 41 mΩ; (**b**) current behavior at different resistors.

Deep discharges of single cells with different load resistors and, consequently, different discharge currents (0.5 … 200 A) to a final clamp voltage of ≈ 0 V were performed. Apart from temperature influences, the resistor over the discharge process can be regarded as almost constant (∆R ≈ 0 … 10%). The voltage drop across the internal resistance of the cell increases due to the low load resistors and the resulting high load currents, resulting in a lower clamp voltage under load. With a resistor of 41 mΩ, for example, an initial current of 80…90 A flows (Fig. 1a,b). Figure 1b shows the corresponding current behaviors at selected resistors (30…74 mΩ).

The voltage plateau in Fig. 1a is explained by the fact that the cathode potential is nearly constant and the anode potential increases only slightly in the range up to approx. 20 Ah^19^. The height of the plateau results from the discharge current. The collapse of the clamp voltage at a discharged charge quantity of about 20 Ah results from the rapid increase of the anode potential (> 3 V vs. Li/Li^+^) with a simultaneous decrease of the cathode potential (≈ 3.5 V vs. Li/Li^+^). When both potentials have reached the same value, the cell is potential-free (U_CV_ = 0 V).

Figure 1a shows that the temperature increases continuously during the discharge at the load resistor of 41 mΩ. The greatest increase is at the point where the clamping voltage collapses rapidly. This rapid temperature rise is associated with the collapse of the SEI^22^. The maximum temperature reached is ≈ 44 °C (Fig. 1a). By contrast, a maximum temperature of about 67 °C was measured when discharging at 13 mΩ. The discharge currents show the same curve as the clamp voltage during discharging. The current is dependent on the value of the resistor (Fig. 1b).

### Opening the cells

The batteries were then opened and the cathode foils analyzed for degradation and copper deposition. The most visually striking finding is that the plastic separator degrades massively during discharge with load resistors ≤ 10 mΩ. In some places, the aluminum oxide coating of the separator adheres so firmly to the cathode that the separator carrier foil can be removed from the aluminum oxide coating. In other places, the separator adheres so firmly to the cathode that it breaks when the separator is pulled off. The gas development during discharging can be followed by inflating the battery. The collapse of the SEI and the resulting gaseous reaction products^22^ cause gas bubbles to form between the foils, which remain between the separator and the cathode foil. No degradation of the separator occurs only at these, typically round or oval places, so that no buildup can be detected there. After opening the cells, none of the foils showed visually recognizable copper deposits on the cathode foil. No deposition of copper independent of the discharge current can be detected by means of REM-EDX analysis. It should be noted that the chemical analysis shows no cobalt, manganese, nickel or copper could be detected in the electrolyte. The EDX analysis of the NMC also shows no measurable influence of the discharge current on the chemical composition of the NMC.

### Discharge of cells connected in series

**Figure 2:**
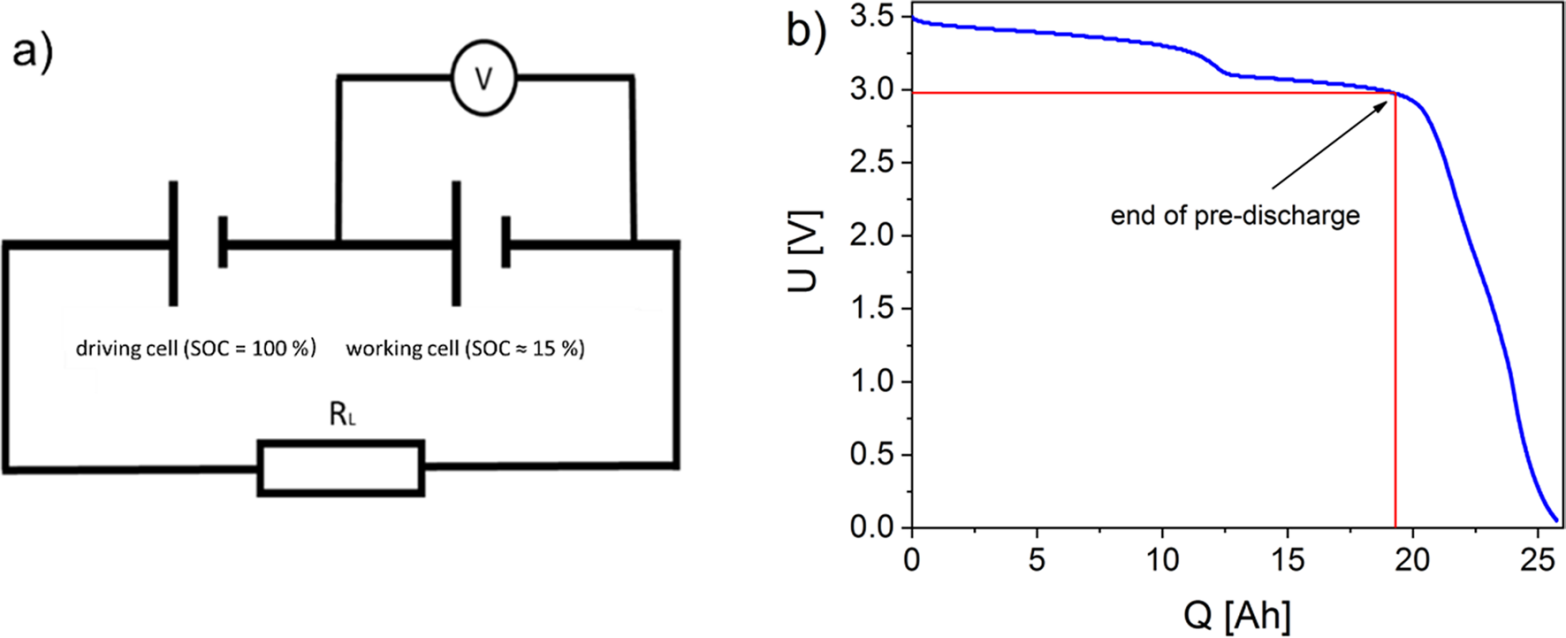
(**a**) Schematic layout of the series connection (SOC Cell 2 <  < SOC Cell 1), (**b**) discharge curve of a single cell at 41 mΩ, red marking, point at which the pre-discharge was stopped for the experiments with a series connection.

Pouch cells with different state-of-charge (SOC_1_ >  > SOC_2_, see Fig. 2) were connected in series and discharged in a further series of experiments. The aim is to simplify the simulation of a battery stack containing batteries with different charge capacities, which can be caused, for example, by production differences (layer quality, capacity differences) or by uneven aging or the failure of individual cells. As a result, the cell with a lower charge capacity is already completely discharged after a shorter discharge time, while the cell with a higher charge capacity still supplies voltage, thus, forcing a current flow in the cell already discharged. The forced current flow causes complex chemical side reactions, such as the almost complete de-intercalation of the anode material, a collapse of the SEI at the anode and, in another process, the dissolution of the copper carrier foil at the carbon anode and the deposition of copper on the cathode^16,20^.

**Figure 3:**
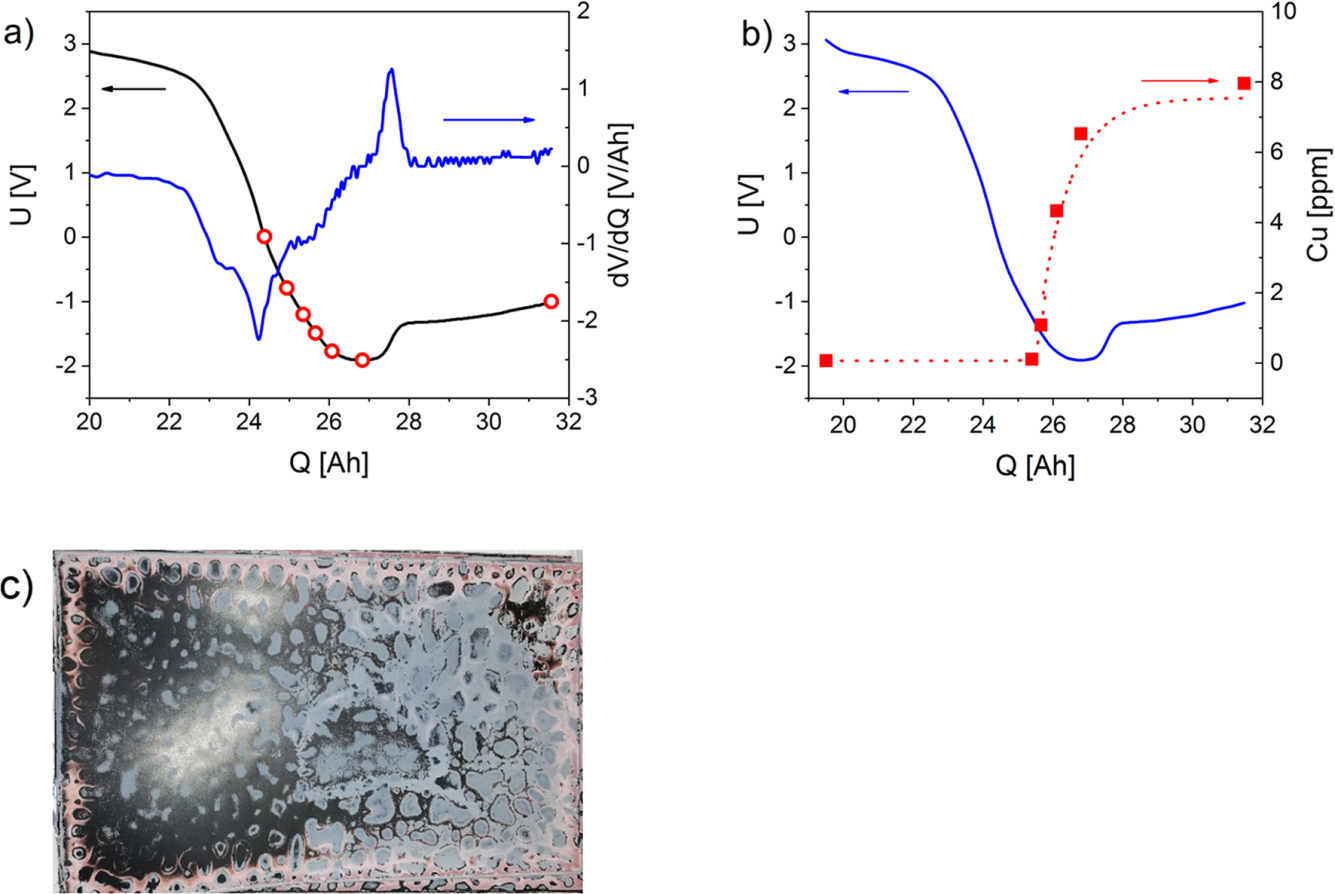
(**a**) Complete discharge curve of a cell during overdischarge at 58 mΩ; markers show the points at which the discharge was stopped and the electrolyte was removed; (**b**) voltage curve of the cells during overdischarge; red dots show the copper concentration in the electrolyte; (**c**) cathode foil with copper deposition (Q = 31.5 Ah).

Figure 3a shows the complete discharge curve and its derivative of a cell during overdischarge. The course of the discharge voltage curves is in very good agreement with the discharge voltage curves determined by Fear et al. The latter concluded from the voltage curves and their 1st and 2nd derivations that the oxidation of the copper foil does not begin until the voltage curve has reached its minimum^16^. In order to provide analytical evidence of which point of the voltage curve marks the dissolution of the copper foil, one cell at a time was discharged to a defined voltage value, the electrolyte was removed and the cell was opened. The copper content in the electrolyte was quantitatively determined by a high-resolution continuum source atomic absorption spectrometer (Table 1). These defined voltage values, each point corresponding to a single cell, are shown as red circles in Fig. 3a and in Table 1. The red squares in Fig. 3b show the copper concentration determined in the electrolyte during overdischarge. The voltage curve (blue line) was drawn in again for illustration. Figure 3c shows a cathode foil (tab. 2, cell 7) with a large area of deposits of copper with the typical “leopard skin” pattern and separator components (white deposits).

**Table 1:** Analysis of electrolyte (w_Cu_, w_Ni,Mn,Co_) and visual inspection of anode (AF) and cathode (CF) foils, x → not present, ✓→ present; U_End_ is the voltage drop across the battery at electrolyte withdrawal, Q is the amount of charge withdrawn from the circuit, cell 7 a.M. after the minimum, LOD_Cu_ = 0.01 mg kg^−1^, n.d. → not detectable.

Cell	1	2	3	4	5	6	7
U_End_/V	0	− 0.8	− 1.2	− 1.5	− 1.77	− 1.91	− 1 a.M
Q/Ah	24.3	24.9	25.3	25.7	26.1	26.8	31.5
w_Cu_/mg kg^−1^	< LOD	< LOD	0.11 ± 0.01	1.08 ± 0.07	4.33 ± 0.15	6.53 ± 0.20	7.96 ± 0.24
w_Ni,Mn,Co_/mg kg^−1^	n.d	n.d	n.d	n.d	n.d	n.d	n.d
Cu on AF	x	x	x	x	✓	x	x
Cu on CF	x	x	x	x	✓	✓	✓

Figure 3a clearly shows that copper is already detectable in the electrolyte at − 1.2 V and, thus, before the voltage minimum is reached. This is remarkable since, according to Fear et al., the electrochemical dissolution of the copper foil should only begin at the voltage minimum (here − 1.91 V)^16^. The amount of charge transferred at − 1.2 V of Q = 25.3 Ah corresponds approximately to the amount of charge that can be withdrawn from a cell that has been individually discharged to 0 V (Fig. 2). Since, from this point on, practically no Lithium-ions are available in the working cell as a charge carrier, the charge flow in the working cell forced by the driving cell leads to the dissolution of the copper of the anode carrier foil and the transport of copper ions to the cathode. At this point, no copper is yet detectable on the cathodes of the opened cell.

**Figure 4:**
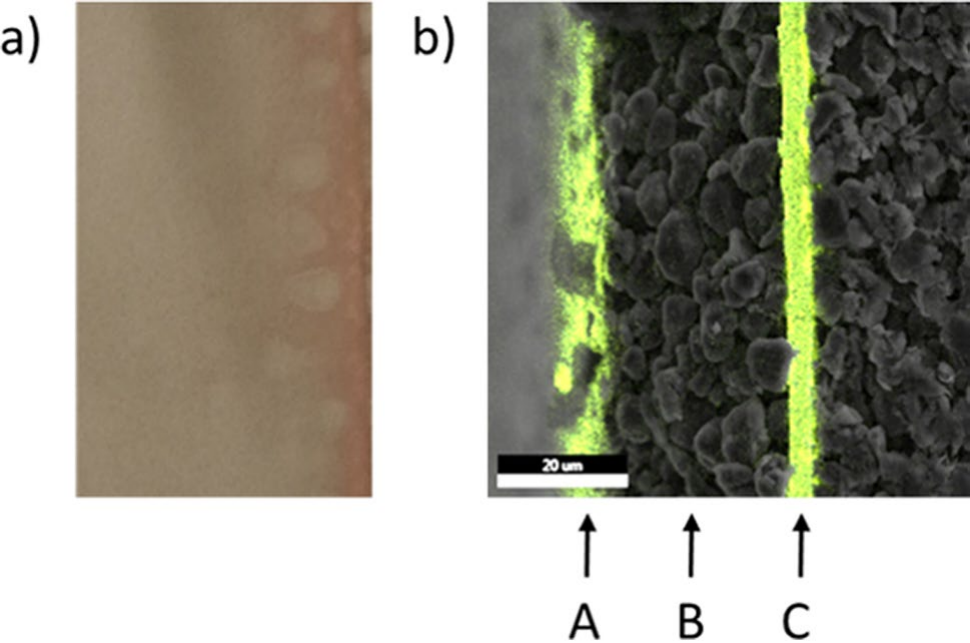
(**a**) Anode foil with copper deposits; (**b**) SEM image with EDX mapping superimposed on the cross-section of the anode foil, A: superficial copper layer, B: graphite layer, C: Cu carrier foil; U = − 1.77 V, Q = 26.1 Ah.

Copper deposits are found both on the cathode and on the anode only at a potential of − 1.77 V (Q = 25.7 Ah) at the working cell. At the same time, an increased concentration of copper ions is detectable in the electrolyte. Copper deposits could also be detected on the surface of the graphite anode (Fig. 4a) only in the cell discharged to − 1.77 V (Q = 26.1 Ah), but not inside the graphite layer (Fig. 4b). Since there is no electron conduction between cathode and anode at this stage, this leads to the assumption that the first copper ions reaching the surface of the graphite are possibly reduced to metallic copper by reactive, reducing decomposition products from the collapse of the SEI. Once these are consumed, the copper deposited on the graphite, which is electronically connected to the copper carrier foil by the graphite, dissolves again in the further course of the discharge of the driver cell. After the collapse of the SEI, a further reduction of copper ions at the anode surface is no longer observed. In the further course of the overdischarge, the deposition of copper only takes place on the cathode surface.

As the discharge proceeds to the voltage minimum, the amount of copper deposited on the cathode foil and the copper concentration in the electrolyte continue to increase. At Q ≈ 27 Ah, the clamping voltage of the work cell rises sharply from − 1.91 V to about − 1.5 V. The cause, according to other authors^14,16,18^, is assumed to be the first copper dendrites growing through the separator and leading to internal short circuits. The first copper dendrites, which grow through the separator and lead to internal short circuits, are suspected of being the cause. Apparently their initial conductivity is very limited, so that although the internal resistance of the cell decreases, further dissolution of the copper foil and further copper deposition on the cathode foil occurs. Thus, at this stage, two conduction mechanisms are present coexisting in parallel. Part of the charge transport takes place through internal short circuits via the first copper dendrites, while parallel charges are transported as a result of the dissolution and deposition of copper. This effect is confirmed by the fact that, despite the decreasing internal resistance, the deposition of copper on the cathode continues to spread and cover it more and more. Finally, as already described by Guo et al., the voltage drop asymptotically approaches 0 V^14^. It follows that the current flow almost completely passes over copper dendrites, which have led to a cell short circuit from the cathode surface across the separator to the graphite layer. The increase in the voltage drop across the working cell at the end of the discharge then corresponds to a decrease in the internal cell resistance due to the electron conduction through the copper dendrites. In their studies, Hendricks et al. found that the deposited copper was non-conductive species^21^. However, they considered it possible that further overdischarge into reversal, as described in this and other work^14,16,18^, could result in deposition of metallic copper.

**Figure 5:**
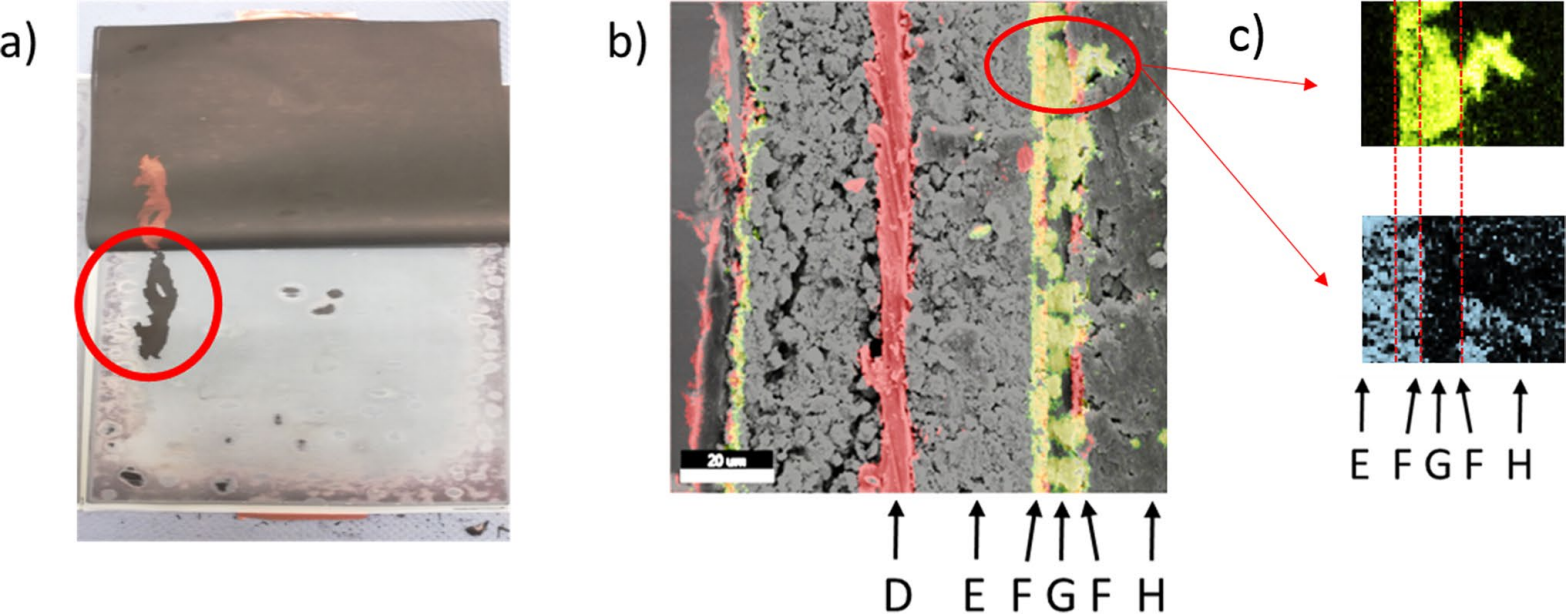
SEM–EDX analysis of cell 7. (**a**) The graphite layer remains on the separator; (**b**) SEM image with EDX mapping superimposed on a cross-section of a cathode foil (red circle on the left) which is connected to the separator and the graphite layer (from left to right) D: Al carrier foil, E: NMC layer, F: Al_2_O_3_ coating of the separator foil (red), G: separator foil penetrated by copper (green), H: graphite layer; (**c**) EDX mapping of a copper dendrite, upper image: copper highlighted in green, lower image: oxygen highlighted in light blue.

Figure 5 shows an example of the top view and cross-section of a cathode foil taken from cell no. 7 after opening. It can be seen that the separator is very firmly attached to the graphite layer in places (Fig. 5a). Figure 5b shows the cross-section of the area marked in Fig. 5a, consisting of an Al carrier foil (D) coated on both sides with NMC (E), plastic separator foil (G) coated on both sides with Al_2_O_3_, and the graphite layer (H). The sharp boundary between the copper layer and the NMC layer shows that the copper is deposited on both sides and flat on the NMC surface of the cathode (Fig. 5b, layer “G”). Furthermore, the plastic membrane of the separator (G) and the Al_2_O_3_ coating of the separator foil (F) are completely penetrated by deposited copper. The original Al_2_O_3_ coating of the separator foil (in red) is still present but seems to be completely penetrated by copper. Figure 5b also shows that copper has grown dendritically from the surface of the NMC coating through the separator and into the carbon coating of the anode (H). In addition, no oxygen is detectable in the deposited copper by EDX (Fig. 5c), which supporting the theory that further overdischarge into reversal results in the deposition of metallic and thus conductive copper^14,16,18,21^.

Finally, it should be noted that the chemical analysis shows that the cathode material is not affected by the discharge processes and no cobalt, manganese or nickel could be detected in the electrolyte (Table 1).

### Temperature characteristics

**Figure 6:**
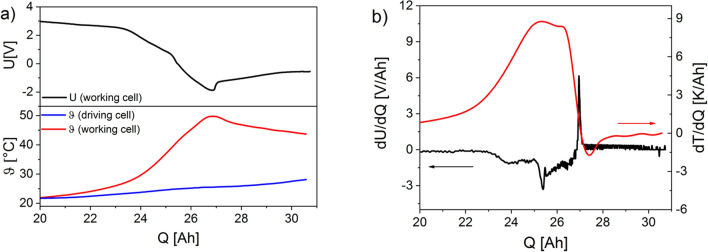
(**a**) Voltage curve and temperature curves during overdischarge of a series connection of the driving cell and the working cell at 58 mΩ. (**b**) Derivation of the voltage curve (black) and derivation of the corrected temperature curve (working cell, red).

Figure 6a shows the voltage curve of the discharge of a working cell at a resistor of 58 mΩ and the temperature curves of the working cell and driver cell. The temperature in the working cell increases only slightly until the end of the capacity expected but very significantly as soon as the voltage drop occurs. This effect becomes obvious because, as shown in Fig. 6b, the strongest increase of the temperature corrected by the waste heat (Newton's cooling law) takes place at the point where the 1st derivative of the voltage function has the local minimum. The maximum temperature of about 50 °C is reached at the voltage minimum of the working cell. The temperature difference between the work cell and the driver cell is, therefore, considerable since the same current flows through both cells. Only when the voltage in the working cell rises again (Fig. 6b, dU/dQ = max), the 1st derivative of the corrected temperature drops to about 0 and the temperature of the cell decreases continuously. It is plausible to assume that the maximum temperatures inside the cell are significantly higher and initiate the degradations there that cause the separator foil to adhere to the surfaces of the electrodes in a virtually non-detachable manner. High temperatures inside the cell are also likely to cause the viscosity of the electrolyte to decrease and the resulting gas bubbles to displace the electrolyte.

## Discussion

### Interpretation of the voltage curve

**Figure 7:**
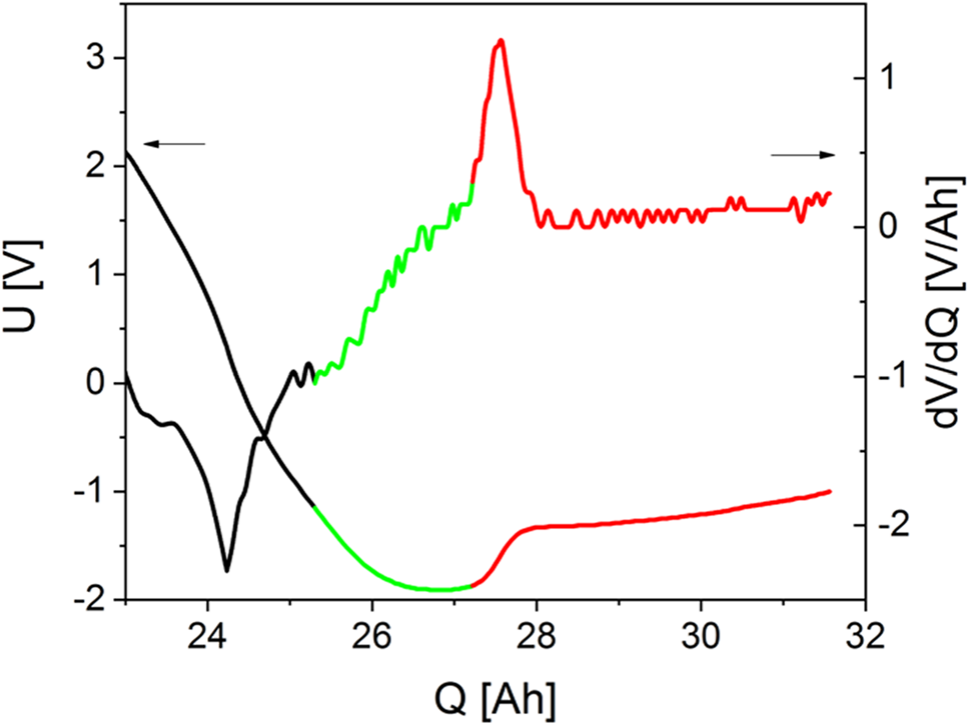
Color coding of the voltage curve and its derivation according to the ongoing processes.

It has been shown that the deep discharge of a single cell does not lead to the dissolution and deposition of copper. If, on the other hand, at least two cells are connected in series in which one cell has a lower charge capacity than the other, deep discharge in this cell causes the copper carrier foil to dissolve and copper to be deposited on the surface of the NMC cathode. If the anode is completely delithiated, no lithium is available for further charge transport. The potential of the series-connected cells of higher charge capacity (SOC > 0) is the driving force to trigger the parasitic process of dissolving the copper in the cell with lower charge capacity, which is discharged earlier (SOC = 0) with further current flow (SOC < 0). Based on the present results, the following interpretation of the discharge curve is suggested (Fig. 7). The black area marks the area of delithiation and breakdown of the SEI, the green area, the copper dissolution and the red area, the internal short circuit through copper dendrites.

The point at which the delithiation is finished as a dominant process is shown as a minimum in the derivative of the voltage curve. This is also the point where the temperature increase is greatest (Fig. 6b). It is assumed that the further, smaller drop in voltage or the subsequent increase in discharge (dV/dQ) is possibly due to the discharge of protic species or the residual water in the electrolyte at the anode^23,24^. This is accompanied by the gas formation, which leads to cell bloating. This process is determinant up to the point where the derivative forms a small plateau (Fig. 7, transition black–green). Following this plateau, the derivative increases more strongly again and the voltage drop proceeds with a smaller increase, which means that the charge transport is less inhibited. In this region, charge transport is carried out by copper ions, although it is still unclear whether this is Cu^+^^16^ or Cu^2+^^14^. The section marked in red is introduced with a steep rise and a subsequent maximum of the derivative function. From this point on, a large number of copper dendrites cause internal short circuits (see Fig. 5) and connect the surfaces of anode and cathode in an electrically conductive manner. Electrochemical processes take on a subordinate role from this moment. As shown in Fig. 6, no further significant heat generation is observed after this stage, which suggests that the electrical work performed is low and the cell behaves approximately as an ohmic resistor.

### Summary

It has been shown that the deep discharge of a single cell does not lead to the dissolution and deposition of copper. If, on the other hand, at least two cells are connected in series in which one cell has a lower charge capacity than the other, deep discharge in this cell causes the copper carrier foil to dissolve and copper to be deposited on the surface of the NMC cathode. If the anode is completely delithiated, no lithium is available for further charge transport. The potential of the series-connected cells of higher charge capacity (SOC > 0) is the driving force to trigger the parasitic process of dissolving the copper in the cell with lower charge capacity, which is discharged earlier (SOC = 0) with further current flow (SOC < 0). By measuring the copper concentration at various points of the discharge curve, analytical proof was provided of the point at which the dissolution of the current collector of the anode begins. Furthermore, it could be shown that the copper concentration in the electrolyte increases rapidly afterwards and converges towards a limit value of about 8 ppm in the investigated interval. Using SEM–EDX mappings, the penetration of the separator and thus the electrical contact between anode and cathode by the copper growing on the cathode surface could be shown. There is no effect on the functional recycling as long as copper ions are present in low concentrations (≤ 1 ppm) in the electrolyte. On the other hand, the deposition of copper on the cathode material over the entire surface leads to significant damage to the NMC recovered since the resulting loss of capacity does not allow for the economic reuse in recycled batteries.

## Methods

### Discharge tests

Pouch cells with cathodes mainly consisting of NMC 622 and a remaining capacity of 20 Ah were used for the experiments. The deep discharge of individual cells was performed with the help of constant resistors (8 mΩ … 5.9 Ω); cell voltage and discharge current were recorded via a data log system (Arduino Uno, time interval of the measuring points = 2 s). The temperature was recorded with a temperature sensor TF-500 type K (PCE Instruments) and with a data logger PCE-T 390 (PCE Instruments) at intervals of 5 s during the deep discharge. Figure 1a shows the test arrangement for the deep discharge of two pouch cells connected in series. Both cells were previously charged to 100% capacity; cell 2 was then discharged via a resistor of 41 mΩ to a clamp voltage (U_CV_) of 3 V, which corresponds to a capacity of 15%. A new pair of pouch cells was used for each deep discharge test in a series connection. If the voltage of the working cell in the deep discharge reached a defined value (U_CV_ = -0.8, -1.2, -1.5, -1.77, -1.91, -1.0 V after the minimum), the test was stopped and the electrolyte was taken from the working cell immediately. The temperature of the working cell was recorded during deep discharge, as described above.

### Electrolyte analysis

The analysis of the electrolyte extracted was carried out using graphite furnace technology with a high-resolution continuum source atomic absorption spectrometer ContrAA 700 (Analytik Jena AG, Jena, Germany). Argon 99.999% (Westfalen AG, Münster, Germany) was used as the inert and purge gas. Characteristic absorption wavelengths were used for the evaluation of the elements nickel (232.0030 nm), cobalt (240.7254 nm), manganese (279.4817 nm) and copper (324.7540 nm).

An equidistant 11-point calibration (0…0.067 mg kg^−1^) in triple determination was carried out regarding the quantification of the copper. The electrolyte sample was manually diluted by a factor of 42…43 (by mass) with deionized water (MilliPore, 18 MΩ cm) and subsequently diluted by a ContrAA 700 autodilution procedure. A calibration was not necessary for Ni, Co and Mn since no element signal could be detected in any of the electrolyte solutions.

### SEM–EDX analysis

All deeply discharged pouch cells were then opened. The morphological and elemental analysis of the dismounted cathode foils were performed using a scanning electron microscope (SEM, Zeiss EVO MA15) equipped with an energy dispersive X-ray analysis (EDX, AMETEK, 20 kV).
